# *REST* rs3796529 Genotype and Rate of Functional Deterioration in Alzheimer’s Disease

**DOI:** 10.14336/AD.2018.0116

**Published:** 2019-02-01

**Authors:** Poyin Huang, Cheng-Sheng Chen, Yuan-Han Yang, Mei-Chuan Chou, Ya-Hsuan Chang, Chiou-Lian Lai, Hsuan-Yu Chen, Ching-Kuan Liu

**Affiliations:** ^1^Department of Neurology, Kaohsiung Municipal Hsiao-Kang Hospital, Kaohsiung Medical University, Kaohsiung, Taiwan.; ^2^Department of Neurology, Kaohsiung Medical University Hospital, Kaohsiung Medical University, Kaohsiung, Taiwan.; ^3^Ph.D. Program in Translational Medicine, Kaohsiung Medical University and Academia Sinica, Taiwan.; ^4^Department of Neurology, Faculty of Medicine, College of Medicine, Kaohsiung Medical University, Kaohsiung, Taiwan.; ^5^Department of Psychiatry, Kaohsiung Medical University, Kaohsiung, Taiwan.; ^6^Department of Neurology, Kaohsiung Municipal Ta-Tung Hospital, Kaohsiung, Taiwan.; ^7^Institute of Statistical Science, Academia Sinica, Taipei, Taiwan.; ^8^Graduate institute of medicine, Kaohsiung Medical University, Kaohsiung, Taiwan.

**Keywords:** genetics, biomarker, dementia, prognosis

## Abstract

Recently, *REST* (RE1-silencing transcription factor) gene has been shown to be lost in Alzheimer’s disease (AD), and a missense minor *REST* allele rs3796529-T has been shown to reduce the rate of hippocampal volume loss. However, whether the *REST* rs3796529 genotype is associated with the rate of functional deterioration in AD is unknown. A total of 584 blood samples from Taiwanese patients with AD were collected from January 2002 to December 2013. The diagnosis of AD was based on the National Institute of Neurological and Communicative Disorders and Stroke and the Alzheimer’s Disease and Related Disorders Association criteria. The allele frequency of rs3796529-T was compared between the AD cohort and 993 individuals from the general population in Taiwan. Kaplan-Meier analysis, the log rank test and a multivariate Cox model were then used to evaluate the association between rs3796529-T and functional deterioration in the AD cohort. The allele frequency of rs3796529-T was significantly lower in the AD cohort compared to the general population cohort (36.82% vs. 40.73%, p=0.029). Kaplan-Meier analysis and the log rank test showed that the AD patients carrying the rs3796529 T/T genotype had a longer progression-free survival than those with the C/C genotype (p=0.012). In multivariate analysis, the rs3796529 T/T genotype (adjusted HR=0.593, 95% CI: 0.401-0.877, p=0.009) was an independent protective factor for functional deterioration. The rs3796529 T/T genotype was associated with slower functional deterioration in patients with AD. This finding may lead to a to better understanding of the molecular pathways involved, and prompt further development of novel biomarkers to monitor AD.

Alzheimer’s disease (AD) is the most common form of dementia worldwide, and it is the leading cause of death in developed countries. Of the top ten causes of death in developed countries, AD is the only disease that cannot be prevented, cured or even slowed [1]. In both developed and developing nations, AD has had an enormous impact on the affected patients, caregivers, and society as a whole.

The last ten years has seen an explosion in the knowledge of genetic variants associated with common diseases [2]. Genetics play a role in susceptibility to all common human diseases including AD, and so identifying genetic biomarkers including single nucleotide polymorphisms (SNPs) and epigenetic markers for AD may facilitate classification of individuals according to drug response, disease progression and prognosis, thereby improving therapeutic outcomes and allowing for personalized management [3]. This concept of “precision medicine” has emerged in recent years as an approach for disease prevention and management that is personalized to an individual’s specific pattern of genetic variability, environment and lifestyle factors [4]. While significant advances in precision medicine have been made for select cancers and a few monogenetic disorders, applications for most other common diseases are still under investigation [5]. To advance the application of precision medicine to a broader spectrum of diseases, governments around the world are starting to launch precision medicine initiatives [1, 5]. However, substantially more research is needed to provide the scientific knowledge required to integrate precision medicine into everyday clinical practice. The application of precision medicine in AD is of great importance since AD is a highly prevalent, clinically and pathologically complex disease. Previous genome-wide association studies (GWAS) have identified numerous SNPs associated with the pathogenesis of AD, including *TTLL7, MMP12, GAB2, PICALM, HRK, RPAP3, ZNF224, PVRL2, BIN1, EPC2, LRAT, MTHFD1L, CD2AP, RELN, EPHA1* and *CLU*. However, most of these SNPs are intronic variations, and thus less medically actionable [6]. Furthermore, most genetic studies on AD have focused on patients with a Caucasian or African-American genetic background, and therefore studies of the behavior of (suspected) genetic risk variants in patients with AD and an Asian genetic background are needed [6].

The *REST* gene (RE1-silencing transcription factor), also known as neuron restrictive silencer factor (NRSF), is a master regulator of neurogenesis and neuronal differentiation. The induction of *REST* is a universal feature in the normal aging of human cortical and hippocampal neurons, and *REST* has been shown to repress genes promoting apoptosis and AD pathology while inducing the expression of stress response genes [7]. *REST* has also been shown to be lost from the nucleus and to appear in autophagosomes together with pathological misfolded proteins in patients with AD and mild cognitive impairment (MCI). In addition, conditional deletion of the *REST* gene in mice brains has been reported to lead to age-related neurodegeneration [7]. *REST* levels during aging are associated with cognitive preservation and longevity, and the activation state of *REST* may distinguish neuroprotection from neurodegeneration in an aging brain. *REST* is considered to protect neurons from amyloid β protein toxicity and oxidative stress, and the *REST* rs3796529 allele has been shown to be a significant protective factor against hippocampal volume loss [7]. However, other studies using a large scale GWAS dataset found that the association between the *REST* rs3796529 allele and susceptibility to AD was not significant in a European population, and that it did not significantly influence human subcortical brain structures [8-10]. Based on these recent findings, *REST* may be considered to be a new gene associated with AD, and the *REST* rs3796529 allele may be considered to be a novel mutation of an AD-associated gene. Since the *REST* rs3796529 allele has been shown to be a significant protective factor against hippocampal volume loss [8], it is reasonable to hypothesize that, from a clinical practice point of view, it may be a potential protective allele for AD, and potentially protect against functional deterioration. Thus, the aim of this study was to identify whether the *REST* rs3796529 genotype is associated with the rate of functional deterioration in AD, which could be useful in clinical practice.

## MATERIALS AND METHODS

### Study Population

Blood samples from 584 patients with AD were collected from the Neurologic Department of Kaohsiung Medical University Hospital from January 2002 to December 2013. The diagnosis of AD was based on the National Institute of Neurological and Communicative Disorders and Stroke and Alzheimer’s Disease and Related Disorders Association (NINCDS-ADRDA) criteria. The patients with AD had to fulfill the following criteria in order to be included in the probable group in this study: (1) a diagnosis of dementia established by clinical and neuropsychological examinations; (2) progressive cognitive impairments present in two or more areas of cognition without conscious disturbance; (3) onset after the age of 40 and before the age of 90 years, mostly after 65 years; and (4) brain CT or MRI studies compatible with AD. The included patients were taking donepezil 5-10 mg/d, rivastigmine 3-6 mg/12 hours, galantamine 8-12 mg/12 hours, or memantine 5-10 mg/12 hours. An annual interview was performed with each patient, and the Clinical Dementia Rating (CDR) score was used to evaluate their functional deterioration. The patients with an increased CDR score during follow-up were defined as having clinical progression. The clinical characteristics including age, gender, education level, ApoE genotype, serial CDR score, baseline stages of AD and medications used for AD were recorded.

Data regarding other disorders of these AD patients including diabetes mellitus, hypertension, malignancies, ischemic stroke, hyperlipidemia, chronic kidney disease and chronic obstructive pulmonary disease were also recorded.

This study was approved by the Institutional Review Board of our hospital, and written informed consent was obtained from all patients or their relatives before participating in this study.

### DNA Extraction and Real-time Quantitative PCR for REST rs3796529 Genotyping

Genomic DNA was extracted from the buffy coat following standard protocols. The *REST* rs3796529 genotype was assayed using quantitative real-time PCR with TaqMan® qPCR assays (Life Technologies, Carlsbad, CA).

### Allele Frequencies in the General Population

Allele frequencies of *REST* rs3796529 in the general population were obtained from the public domain including the Exome Aggregation Consortium (ExAC) for East Asians (4321 cases) [11], 1000 Genomes for East Asians (504 cases) and Chinese (208 cases) [12], and the Taiwan Biobank for Taiwanese (993 cases) [https://www.twbiobank.org.tw/new_web/index.php].

### Statistical Analysis

The chi-square test or ANOVA was used to compare differences between groups for categorical or continuous variables, respectively. The Kaplan-Meier method was used to estimate overall progression, and the difference between curves was tested using the log-rank test. Multivariate Cox proportional hazards regression analysis with covariates including age, gender, education and baseline CDR was used to evaluate the independent prognostic factors for AD cognitive decline. Variables with more than 10% missing data were not included in the multivariate analysis (medication and ApoE genotype). All tests were two-sided, and p values < 0.05 were considered to be statistically significant.

### RESULTS

**Figure 1.**
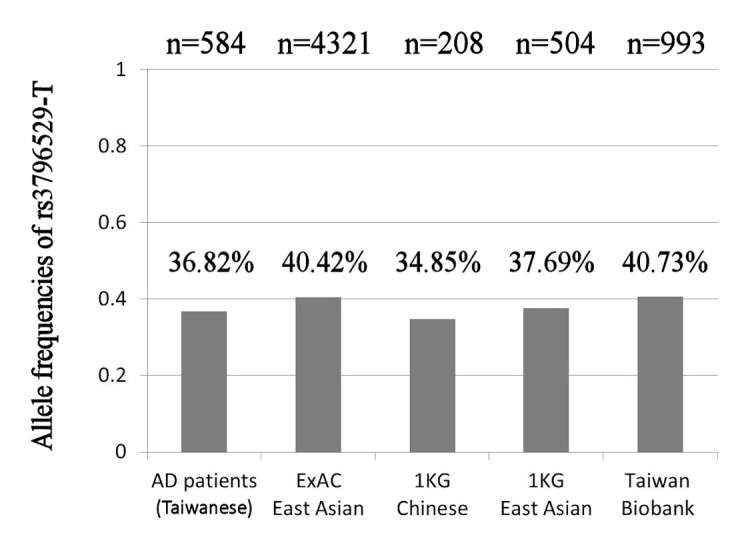
Bar chart of allele frequencies of rs3796529-T in patients with AD and East Asian general populations.

A total of 584 patients with AD were enrolled in this study. The median progression time was 2 years (range from 0.5 to 10 years). The allele frequencies of *REST* rs3796529-T were 36.82% in the patients with AD, 40.42% in East Asians from ExAC, 34.85% in Han Chinese from 1000 Genomes, 37.69% in East Asians from 1000 Genomes, and 40.73% in Taiwanese from the Taiwan Biobank (Fig. 1). The odds ratios of the allele frequency of *REST* rs3796529-T were 1.164 (95% confidence interval (CI): 1.026-1.321, p=0.018) for ExAC East Asians, 0.918 (95% CI: 0.727-1.161, p=0.475) for 1000 Genomes Han Chinese, 1.039 (95% CI: 0.872-1.236, p=0.671) for 1000 Genomes East Asians, and 1.180 (95% CI: 1.017-1.369, p=0.029) for Taiwan Biobank Taiwanese compared to the Taiwanese patients with AD (Table 1).

**Table 1:** Allele frequencies, allele numbers and odds ratios of rs3796529-T in patients with AD and East Asian general populations.

*REST* rs3796529	Total AN	T allele AN (%)	Odds ratio (95% CI)	p value
AD patients (n=584)	1168	430 (36.82%)		
ExAC East Asian	8642	3493 (40.42%)	1.164 (1.026-1.321)	0.018
1000 Genomes Han Chinese	416	145 (34.85%)	0.918 (0.727-1.161)	0.475
1000 Genomes East Asian	1008	380 (37.69%)	1.039 (0.872-1.236)	0.671
Taiwan Biobank Taiwanese	1986	402 (40.73%)	1.180 (1.017-1.369)	0.029

Data are presented as n (%); p value by χ2. AD=Alzheimer’s disease; AN=allele number.

In addition, 239 AD patients had *REST* rs3796529- C/C, 260 had *REST* rs3796529- C/T and 85 had *REST* rs3796529- T/T. Of the East Asians from ExAC, 720 had *REST* rs3796529- T/T, 2053 had *REST* rs3796529- C/T, and 1548 had *REST* rs3796529- C/C. Of the Han Chinese from 1000 Genomes, 26 individuals had *REST* rs3796529- T/T, 93 had *REST* rs3796529- C/T, and 89 had *REST* rs3796529- C/C. Of the East Asians from 1000 Genomes, 74 individuals had *REST* rs3796529- T/T, 232 had *REST* rs3796529- C/T, and 198 had *REST* rs3796529- C/C. Of the Taiwanese from the Taiwan Biobank, 164 individuals had *REST* rs3796529- T/T, 481 had *REST* rs3796529- C/T, and 348 had *REST* rs3796529- C/C. Thus, the carrier frequency of *REST* rs3796529-T was 59.08% for the AD cohort, 64.17% for the ExAC East Asians, 57.21% for the 1000 Genomes Han Chinese, 60.71% for the 1000 Genomes East Asians, and 64.95% for the Taiwan Biobank Taiwanese.

The patients with AD were then categorized into *REST* rs3796529 C/C, *REST* rs3796529 C/T and *REST* rs3796529 T/T groups. The basic clinical characteristics of these three groups are shown in Table 2. There were no significant differences in age, gender, education level, ApoE genotype, baseline CDR, baseline stages of AD or medications used for AD, among the three *REST* rs3796529 genotype groups.

**Figure 2.**
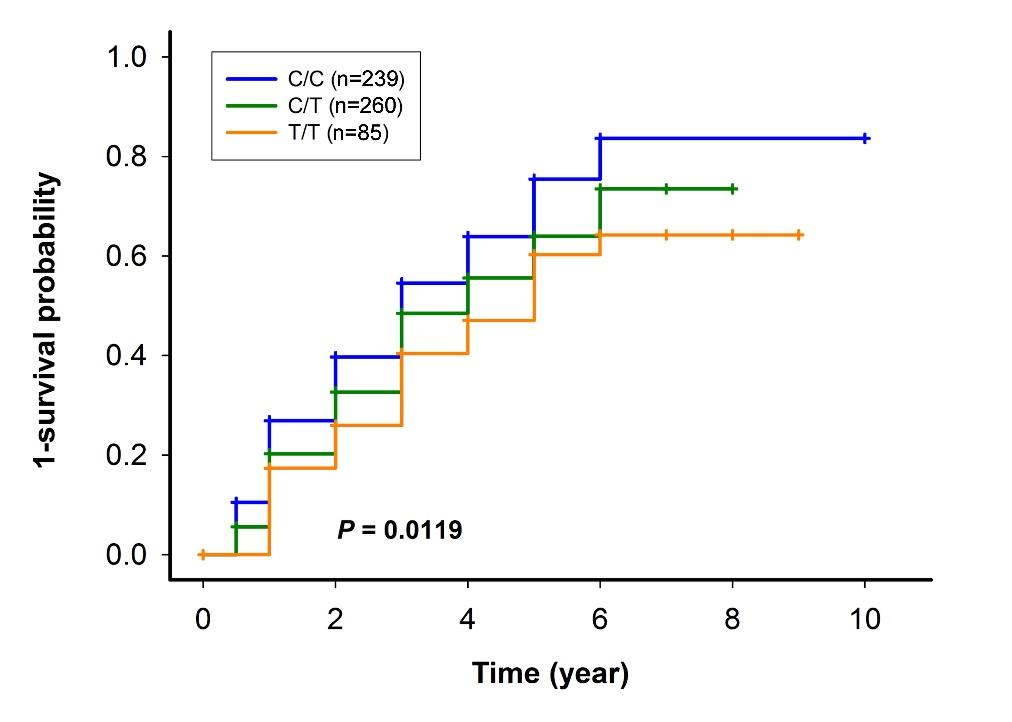
The 1-survival probability curve of the progression of AD for *REST* rs3796529 C/C was above that of *REST* rs3796529 C/T and T/T; p value according to the log-rank test.

For AD functional deterioration, the results of survival analysis showed that the progression probability curves were high, median, and low for *REST* rs3796529 C/C, C/T, and T/T, respectively (p=0.012) (Fig. 2). In addition, the adjusted hazard ratio (HR) of *REST* rs3796529 T/T was 0.593 (95% CI: 0.401-0.877, p=0.009), and *REST* rs3796529 T/T was an independent prognostic factor for AD functional deterioration, along with age (adjusted HR=0.983, 95% CI: 0.967-0.998, p=0.031) and baseline CDR (adjusted HR=0.590, 95% CI: 0.451-0.773, p<0.001) (Table 3).

For more information, data regarding other disorders of these AD patients including diabetes mellitus, hypertension, malignancies, ischemic stroke, hyperlipidemia, chronic kidney disease and chronic obstructive pulmonary disease were shown in Supplementary Table 1. Among these disorders, hypertension (p=0.007) and ischemic stroke (p=0.024) were associated with rs3796529 genotype. However, when adding hypertension and ischemic stroke as covariates in multivariate Cox proportional hazards regression analysis, the results remained unchanged (Supplementary Table 2). *REST* rs3796529 T/T was still an independent prognostic factor for AD functional deterioration (adjusted HR=0.574, 95% CI: 0.386-0.853, p=0.006) while hypertension and ischemic stroke were not (Supplementary Table 2).

**Table 2:** Basic characteristics of the AD patients categorized by rs3796529 genotype.

AD patients (n=584)	C/C (n=239)	C/T (n=260)	T/T (n=85)	p value
Age (years) (n=559)	79.10±8.10 (n=228)	79.49±7.68 (n=249)	78.70±7.64 (n=82)	0.745
Gender (Male) (n=566)	29.9% (n=69)	29.4% (n=74)	28.9% (n=24)	0.985
Education (years) (n=540)	5.71±4.87 (n=223)	6.13±4.78 (n=240)	6.14±5.00 (n=77)	0.599
APOE E4 carrier (n=274)	32.4% (n=36)	38.1% (n=43)	30.0% (n=15)	0.525
Baseline CDR (0.5 or 1) (n=550)	80.5% (n=178)	75.0% (n=186)	80.2% (n=65)	0.305
Baseline stages of AD				0.140
Early (CDR=0.5 or 1)	80.5% (n=178)	75% (n=186)	80.2% (n=65)	
Middle (CDR=2)	19% (n=42)	24.6% (n=61)	17.3% (n=14)	
Late (CDR=3)	0.5% (n=1)	0.4% (n=1)	2.5% (n=2)	
(n=550)	(n=221)	(n=248)	(n=81)	
Medication- Donepezil	69.1%	71.3%	73.7%	0.476
Rivastigmine	24.2%	18.4%	22.4%	
Galantamine	4.8%	6.7%	2.6%	
Memantine	1.9%	3.6%	1.3%	
(n=506)	(n=203)	(n=227)	(n=76)	

Data are presented as mean ± standard deviation or n (%); p value by χ2 or two-tailed ANOVA. AD=Alzheimer’s disease

### DISCUSSION

The aberrant expression of the *REST* gene has been reported in a broad range of diseases including various tumors, diabetes mellitus and heart diseases [13-15]. However, the role of the *REST* gene in neurodegeneration is less clear [13]. Increasing evidence has shown that *REST* plays a pivotal role in human neurodegeneration, and many direct *REST* target genes encoding proteins crucial for nervous system development have been identified [16]. *REST* has also been widely detected in brain regions, and it has been shown to be highly expressed in neurons and to increase with aging in the human brain. Altered *REST* gene expressions have also been associated with deficient brain function such as in neurodegenerative diseases, mental disorders, brain tumors, neurobehavioral disorders, brain injuries and stroke [17-22]. The loss of its expression and cytoplasmic translocation have been hypothesized to play a pivotal role in several human dementias including AD, frontotemporal dementia and dementia with Lewy bodies [17, 21]. In these neurodegenerative diseases, *REST* has been shown to be lost from the nucleus and to appear in autophagosomes together with pathological misfolded proteins including Ab, phosphorylated tau, TDP-43 and alpha-synuclein. These findings represent a common pathogenic mechanism that links altered proteostasis to aberrant gene expression [7]. Thus, *REST* is considered not only to be a classical repressor necessary to maintain normal neurogenesis, but also to be a fundamental protector against neurodegeneration and possibly an important target for neurodegenerative diseases such as AD [21-25].

The *REST* gene has only very recently been directly associated with AD. However, the association between the *REST* rs3796529 allele and AD is still unclear as the results of previous studies have been inconsistent [7-10]. In 2014, the *REST* gene was found to be involved in neurogenesis and neuronal differentiation, and also that the expression of the *REST* gene may underlie protection against neurodegenerative processes in AD [7]. In 2015, Nho et al used a whole exome sequencing method to identify potential variants associated with hippocampal loss in MCI, and the *REST* missense variant rs3796529 was identified exclusively in subjects with slow hippocampal volume loss [8]. Their study was the first to show that the minor T allele of rs3796529 confers a protective effect on hippocampal loss [8]. Other researchers then used a large scale GWAS dataset to investigate the potential association between the *REST* rs3796529 variant and AD. Though they found that the rs3796529 variant T allele seemed to be associated with reduced AD risk (beta = -0.0254 and standard error = 0.0203), but the association between *REST* rs3796529 and

AD susceptibility is not significant (p = 0.2101). Thus, they concluded that *REST* rs3796529-T did not confer susceptibility to AD in a European population. They also found that the rs3796529 variant did not confer a significant effect on hippocampal loss, and suggested that further studies were needed to clarify these findings [9-10].

In this study, the frequency of missense allele *REST* rs3796529-T was intermediate in the East Asian cohort, ranging from 34.85% to 40.73%, compared to 36.82% in the Taiwanese cohort with AD, which was statistically lower than that of the Taiwanese general population cohort. This finding indirectly implies that *REST* rs3796529-T may be a protector against AD from the viewpoint of population genetics, since the allele frequency was lower when compared within the same ethnic group. Further analysis within the AD cohort showed that patients with *REST* rs3796529-T had a lower rate of functional deterioration. Therefore, from a clinical point of view and taking the results of previous *in vitro* and animal-based studies into consideration, *REST* rs3796529-T can be considered to be a potential protective allele for AD. Given that *REST* rs3796529 is a missense mutation, the potential mechanism of the *protective effect in progression* to AD could be explained by the results from a previous study, in which *REST* rs3796529 was a significant protective factor against hippocampal volume loss [8]. However, AD is a heterogeneous and complex disease. Due to the small risk estimates of the genetic variants, it may be difficult to use a SNP as a diagnostic or predictive marker in everyday clinical practice. A combination of several potential genes may help to achieve a higher level of accuracy, since the small effect of many potential genes can be added to improve the overall predictive power. Its use lies primarily in a better understanding of the molecular pathways involved, and this may contribute to the discovery of AD mechanisms and novel candidate therapeutic targets. The findings of this study support that the missense allele rs3796529-T, located within exon 4 of *REST* on chromosome 4, may confer a protective effect against AD, since this variant was identified to be an independent prognostic factor for AD functional deterioration with an adjusted HR of 0.593 (95% CI: 0.401-0.877, p=0.009). Furthermore, in clinical practice, there is currently no consensus on the most appropriate interval for repeating neuropsychological examinations to evaluate treatment, and more frequent neuropsychological testing alone is not effective in identifying individuals prone to faster functional deterioration. Since the patients without rs3796529-T had a higher probability of developing functional deterioration, molecular studies may be able identify the individuals at higher risk who should be more closely monitored, and such studies may also contribute to the further development of novel biomarkers to monitor the disease.

**Table 3:** Adjusted hazard ratios of risk factors in AD progression.

Variable	Hazard ratio (95% CI)	p value
Male	0.868 (0.639-1.181)	0.368
Age (years)	0.983 (0.967-0.998)	0.031
Education (years)	1.016 (0.988-1.045)	0.257
Baseline CDR score	0.590 (0.451-0.773)	<0.001
REST rs3796529 genotype (C/C as reference)		
C/T	0.793 (0.607-1.035)	0.088
T/T	0.593 (0.401-0.877)	0.009

The main limitations of this study are that the assumption that rs3796529-T had a protective effect was made based on the results of a previous study [7]. Thus, this article lacks information about the possible functional consequences of this particular missense variant, and how this would relate to the suggested protective effect of the rs3796529-T allele on functional deterioration in AD patients. We also found that hypertension and ischemic stroke were associated with rs3796529 genotype. However, based on current literature, no other disorders or diseases were shown to be associated with *REST* rs3796529 except AD, mild cognitive impairment and neurodegeneration. Thus, the exact mechanism of the association between hypertension, ischemic stroke and rs3796529 genotype is unclear. This issue also needs to be addressed by further studies.

### Conclusions

The results of this study demonstrated that the rs3796529 genotype T/T was associated with slower functional deterioration in patients with AD. This finding may lead to a better understanding of the molecular pathways involved, and lead to the further development of novel biomarkers to monitor AD.

## Supplementary Materials

The Supplemenantry data can be found online at: www.aginganddisease.org/EN/10.14336/AD.2018.0116


