# Supplementary material for: REST rs3796529 Genotype and Rate of Functional Deterioration in Alzheimer’s Disease
Source: Aging Dis. 2019 Feb 1;10(1):94–101. doi: 10.14336/AD.2018.0116 (PMC6345341; doi:10.14336/AD.2018.0116)
Supplement: Supplementary file 1 [file AD-10-01-94-s.pdf]

SUPPLEMENTARY DATA

REST rs3796529 Genotype and Rate of Functional Deterioration in Alzheimer's Disease

Poyin Huang[1,2,3,4] Cheng-Sheng Chen[5], Yuan-Han Yang[6], Mei-Chuan Chou[6], Ya-Hsuan Chang[7], Chiou-Lian Lai[1, *], Hsuan-Yu Chen[3,7,8, *], Ching-Kuan Liu[2,*]

[1]Department of Neurology, Kaohsiung Municipal Hsiao-Kang Hospital, Kaohsiung Medical University, Kaohsiung, Taiwan. [2]Department of Neurology, Kaohsiung Medical University Hospital, Kaohsiung Medical University, Kaohsiung, Taiwan. [3]Ph.D. Program in Translational Medicine, Kaohsiung Medical University and Academia Sinica, Taiwan. [4]Department of Neurology, Faculty of Medicine, College of Medicine, Kaohsiung Medical University, Kaohsiung, Taiwan. [5]Department of Psychiatry, Kaohsiung Medical University, Kaohsiung, Taiwan. [6]Department of Neurology, Kaohsiung Municipal Ta-Tung Hospital, Kaohsiung, Taiwan. [7]Institute of Statistical Science, Academia Sinica, Taipei, Taiwan. [8]Graduate institute of medicine, Kaohsiung Medical University, Kaohsiung, Taiwan.

© 2018. Huang P et al. Published online at http://www.aginganddisease.org/EN/10.14336/AD.2018.0116

SUPPLEMENTARY DATA

Supplementary Table 1. Comorbidities of the AD patients categorized by rs3796529 genotype.

AD patients (n=566)	C/C (n=235)	C/T (n=250)	T/T (n=81)	p value
Diabetes mellitus (n=566)	23.4% (n=55)	18.0% (n=45)	21.0% (n=17)	0.339
Hypertension (n=566)	40.9% (n=96)	48.0% (n=120)	28.4% (n=23)	0.007
Malignancies (n=566)	8.1% (n=19)	7.6% (n=19)	8.6% (n=7)	0.951
Ischemic stroke (n=566)	3.0% (n=7)	8.4% (n=21)	3.7% (n=3)	0.024
Hyperlipidemia (n=566)	2.6% (n=6)	2.8% (n=7)	1.2% (n=1)	0.729
Chronic kidney disease (n=566)	1.3% (n=3)	0.8% (n=2)	0% (n=0)	0.561
COPD (n=566)	1.3% (n=3)	1.2% (n=3)	1.2% (n=1)	0.997

Data are presented as n (%); p value by $\chi 2$. AD=Alzheimer's disease. COPD=Chronic obstructive pulmonary disease.

Supplementary Table 2. Adjusted hazard ratios of risk factors in AD progression.

Variable	Hazard ratio (95% CI)	p value
Male	0.850 (0.623-1.159)	0.305
Age (years)	0.983 (0.967-0.998)	0.03
Education (years)	1.018 (0.989-1.047)	0.225
Baseline CDR score	0.582 (0.444-0.764)	<0.001
REST rs3796529 genotype (C/C as reference)		
C/T	0.785 (0.600-1.028)	0.079
T/T	0.574 (0.386-0.853)	0.006
Hypertension	1.150 (0.892-1.483)	0.280
Ischemic stroke	0.818 (0.495-1.351)	0.433

Variables with more than 10% missing data were not added in the multivariate analysis (medication and ApoE genotype).
AD=Alzheimer's disease; CI=confidence interval; CDR= Clinical Dementia Rating

© 2018. Huang P et al. Published online at http://www.aginganddisease.org/EN/10.14336/AD.2018.0116
